# Radiomics and artificial neural networks modelling for identification of high-risk carotid plaques

**DOI:** 10.3389/fcvm.2023.1173769

**Published:** 2023-07-06

**Authors:** Chengzhi Gui, Chen Cao, Xin Zhang, Jiaxin Zhang, Guangjian Ni, Dong Ming

**Affiliations:** ^1^ Academy of Medical Engineering and Translational Medicine, Tianjin University, Tianjin, China; ^2^ Tianjin Huanhu Hospital, China; ^3^ Institute of Biomedical Engineering, Chinese Academy of Medical Sciences and Peking Union Medical College, Tianjin, China; ^4^ School of Medical Science and Engineering, Tianjin University, Tianjin, China

**Keywords:** prognosis, MRI image analysis, radiomics, machine learning, deep learning, stroke risk assessment

## Abstract

**Objective:**

In this study, we aimed to investigate the classification of symptomatic plaques by evaluating the models generated via two different approaches, a radiomics-based machine learning (ML) approach, and an end-to-end learning approach which utilized deep learning (DL) techniques with several representative model frameworks.

**Methods:**

We collected high-resolution magnetic resonance imaging (HRMRI) data from 104 patients with carotid artery stenosis, who were diagnosed with either symptomatic plaques (SPs) or asymptomatic plaques (ASPs), in two medical centers. 74 patients were diagnosed with SPs and 30 patients were ASPs. Sampling Perfection with Application-optimized Contrasts (SPACE) by using different flip angle Evolutions was used for MRI imaging. Repeated stratified five-fold cross-validation was used to evaluate the accuracy and receiver operating characteristic (ROC) of the trained classifier. The two proposed approaches were investigated to train the models separately. The difference in the model performance of the two proposed methods was quantitatively evaluated to find a better model to differentiate between SPs and ASPs.

**Results:**

3D-SE-Densenet-121 model showed the best performance among all prediction models (AUC, accuracy, precision, sensitivity, and F1-score of 0.9300, 0.9308, 0.9008, 0.8588, and 0.8614, respectively), which were 0.0689, 0.1119, 0.1043, 0.0805, and 0.1089 higher than the best radiomics-based ML model (MLP). Decision curve analysis showed that the 3D-SE-Densenet-121 model delivered more net benefit than the best radiomics-based ML model (MLP) with a wider threshold probability.

**Conclusion:**

The DL models were able to accurately differentiate between symptomatic and asymptomatic carotid plaques with limited data, which outperformed radiomics-based ML models in identifying symptomatic plaques.

## 1. Introduction

Many recent studies have shown that vulnerable plaques are generally associated with a high risk of cerebral infarction, and the identification of vulnerable plaques by assessing plaque components is becoming increasingly crucial (1). Characteristic plaque components, such as intraplaque hemorrhage (IPH) and lipid-rich necrotic core (LRNC), are highly associated with ischemic cerebrovascular events and are usually referred to as SPs (2). Early identification of SPs may facilitate prognosis and thereby mitigate adverse outcomes of ischemic cerebrovascular events. In the literature, CT and ultrasound-based texture analysis of plaques has been used to differentiate SPs from ASPs (3). Compared to CT and ultrasound, 3D-HRMRI has a good resolution in soft tissue, and the combination of multiple contrast levels provides more valuable information in clinical practice. Recently, many researchers have introduced ML based technologies to process high dimensional data from HRMRI. Le et al. (4) found that 3D imaging models have better robustness and predictive accuracy than 2D imaging models. HRMRI can accurately identify the composition of plaques; however, the small size of plaques and the lack of histological validation make clinical application challenging (5, 6). Evaluation of plaque characteristics in symptomatic patients showed that fibrous cap thickness, the presence of IPH, and the size of an LRNC can be imaging biomarkers of ischemic events (7). However, images contain much more information than can be visualized or quantified by simple manual measurements.

Recently, the emergence of HRMRI acquisition and artificial intelligence technologies provides opportunities to transform HRMRI image information into quantitatively mineable data. One of the key risk factors for stroke is plaque stability, and many studies have focused on the non-invasive identification of symptomatic plaques to guide treatment strategies (8). The identification of imaging features of SPs via visual assessment of radiology professionals is the most intuitive, but it requires years of professional training and is partially subjective. In the study reported by Chen et al. (7), the AI model (*p* = 0.0003) performed better than visual assessment model (*p* = 0.021). Researchers prefer to build AI models because they offer several advantages over traditional methods. These models can evaluate large amounts of data quickly and accurately, automate tedious tasks, reduce the potential for human error, and provide objective insights.

Radiomics-based image analysis is proposed to extract and analyze a large number of quantitative features from regions of interest (ROIs), which are believed to reflect the imaging phenotype of carotid plaques. Radiomics-based ML models are an important tool for differentiating SPs from ASPs (9, 10). Combining radiomics analysis with classical ML and integrated learning algorithms is an emerging technology.

However, high-throughput radiomics analysis is limited by the manual delineation of carotid plaque boundaries, which is time-consuming and poorly reproducible in creating ROIs. DL algorithms are considered to be more advanced ML techniques and are used in many research areas. DL is based on various artificial neural networks that learn effective features from image data without delineating carotid plaque boundaries, which can greatly reduce the time for HRMRI image pre-processing (11). For image analysis, DL technologies have proven to be effective in disease classification as well as localization and segmentation of lesions, and these techniques have shown superior accuracy and efficiency in diagnostic and image analysis tasks compared to traditional methods (12).

The accurate recognition of carotid plaques using deep learning is challenged by limitations in the dataset, overfitting, and redundant computations. To optimize feature extraction and reduce unnecessary computations, we chose DenseNet, which has a unique connectivity pattern that effectively mitigates gradient disappearance. Additionally, SENet enhances the relevant feature channels while suppressing the less useful ones, enabling adaptive recalibration and improving accuracy. We integrated SENet (13) with DenseNet to extract useful information and achieve high accuracy in recognizing carotid plaques.

The purpose of this study was to investigate the feasibility of discriminate SPs and ASPs based on MRI images. The discrimination models were generated through two approaches in this paper, a radiomics-based ML and an end-to-end DL approach.

## 2. Materials and methods

**Figure 1:**
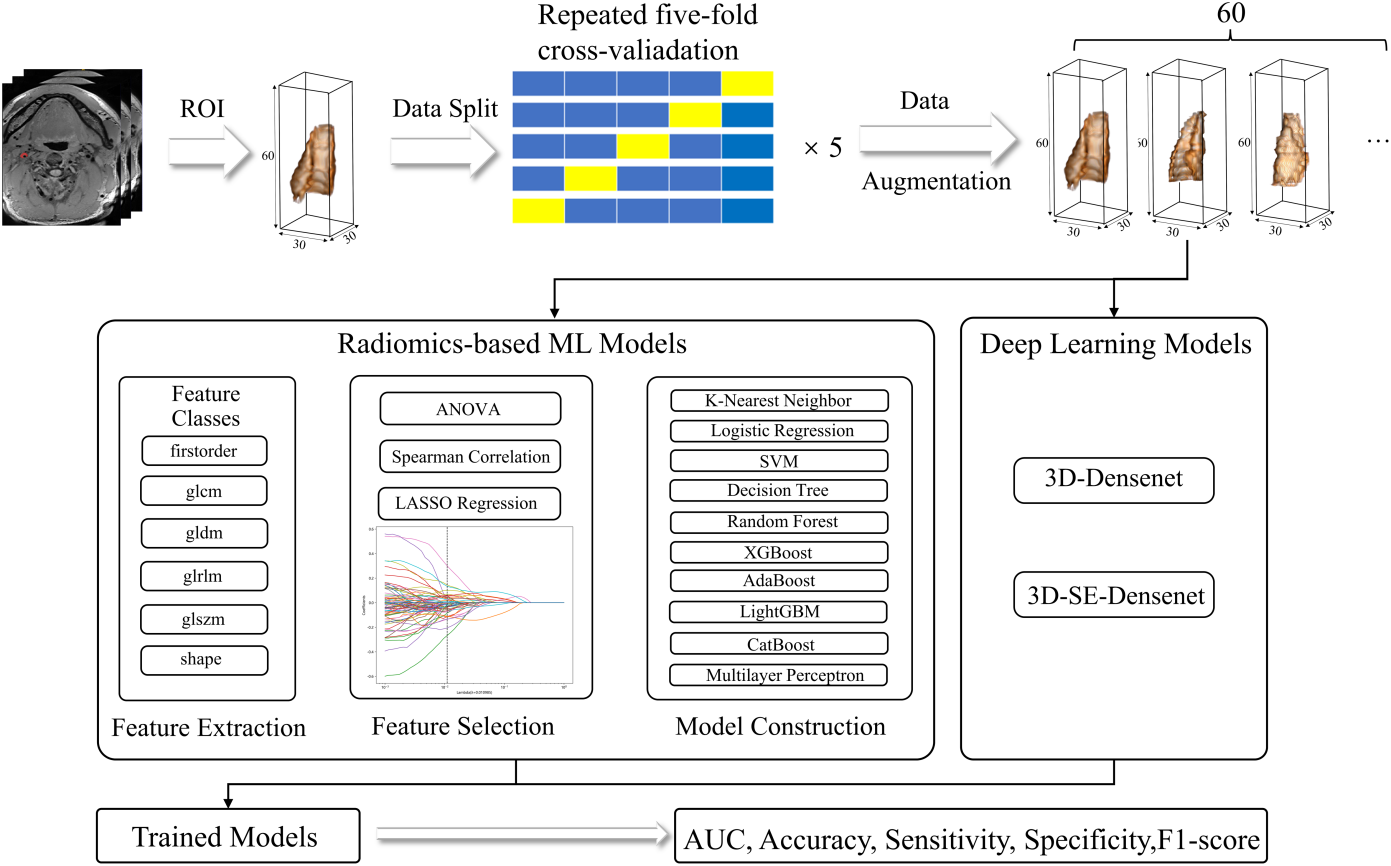
Radiomics and DL pipelines. The two approaches were developed separately, and the performance was evaluated based on AUC, accuracy, sensitivity, specificity, and F1-score.

The overall process pipelines were summarized in Figure 1.

### 2.1. Participant recruitment

This study was conducted in accordance with the Declaration of Helsinki and ethics approval was obtained from the local institutional ethics review board. HRMRI data of 195 patients with carotid plaques were collected in Tianjin Huanhu Hospital and Tianjin First Central Hospital from December 2016 to April 2021. Participants provided informed written consent for retrospective data analysis. The principles for the inclusion and exclusion criteria were set in accordance with the literature (7).

Inclusion criteria were set as (a) Patients had an acute ischemic stroke within the past 7 days, whose corresponding unilateral infarction was confined to a single carotid region as defined by diffusion-weighted imaging (14); (b) Patients with symptom duration ≤24 h met the WHO definition of transient ischemic attack but had documented acute ischemic infarction; (c) carotid lumen stenosis >30% (15).

Exclusion criteria were set as (a) patients with ≥70% carotid stenosis; (b) cardiogenic stroke; (c) patients with bilateral infarcts or clinical signs due to bilateral carotid plaques; and (d) other causes, such as MRI images missing some slice data. 91 patients were excluded from the study.

Ischemic stroke can be caused by both the characteristics of carotid plaque and degree of stenosis. In patients with less than 30% carotid stenosis, the carotid plaque is small and still in the formation stage, which is not likely related to the current ischemia status of the participant. Embolism in patients who have had an ischemic stroke may be originated from an embolus elsewhere in the body other than carotid. Therefore, patients with less than 30% carotid stenosis should be excluded to prevent interference from other factors (15). On the other hand, patients with carotid artery stenosis greater than 70% may experience ischemic stroke due to insufficient blood supply, rather than the characteristics of the carotid plaque. Therefore, patients with carotid artery stenosis greater than 70% should also be excluded in this study (16). Therefore, in this study, we sought to establish a quantitative imaging biomarker to identify SPs and ASPs in 30%–70% carotid stenosis.

All patients were divided into SPs and ASPs groups. The detailed criteria for the diagnosis of SPs are: patients with regions of ADC <620 × 10^−6^ mm^2^/s (CST-ADC) (17) or Tmax >6 s mismatch volume (penumbra volume–infarct volume) of 15 ml or more (18). The rest of the carotid plaque is ASPs.

Finally, we utilized high-resolution magnetic resonance imaging (HRMRI) data from 104 patients with carotid artery stenosis, who were diagnosed with either symptomatic plaques (SPs) or asymptomatic plaques (ASPs), in two medical centers. 74 patients were diagnosed with SPs and 30 patients were ASPs.

In our study, we focused on patients with 30%–70% carotid artery stenosis. Previous studies have shown that the stroke risk is consistently 2–3 times higher for 70%–99% distal stenosis compared to 50%–69% stenosis. Compared to 70%–99% stenosis, 50%–69% stenosis is not a high risk and major factor for SPs (16). In contrast, in patients with <30% carotid stenosis, ischemic stroke may not originate from symptomatic plaque in the carotid artery. Therefore, we sought to establish a quantitative imaging biomarker to identify SPs and ASPs in 30%–70% carotid stenosis.

### 2.2. Data split

In order to address the limitation of a small sample size, we performed a repeated stratified five-fold cross-validation approach. This was done five times to ensure robustness of the results and obtain more reliable mean and standard values for the classification metrics. This approach was applied to both machine learning and deep learning methods in our study.

### 2.3. Magnetic resonance imaging data

The MRI imaging equipment of Tianjin Huanhu Hospital and Tianjin I Central Hospital are of the same type. All imaging data were acquired from two 3-T MRI systems (MAGNETOM Prisma, Siemens Healthcare, Erlangen Germany) with a 64-channel integrated head/neck coil. The imaging protocol included SPACE, DWI, and DSC-PWI. For the SPACE sequencing, the repetition time was set to 700 ms, the echo time was 12 ms, and the slice thickness was 1.0 mm. DWI images were acquired using a spin-echo type echo-planar (SE-EPI) sequence with b values as 0 and 1,000 s/mm^2^. In addition, apparent diffusion coefficient (ADC) maps were calculated from the diffusion scan raw data in a pixel-by-pixel manner. For the parameters of DWI, the repetition time was set to 2,900 ms; the echo time was set to 73 ms, the field of view was set to 240 × 240 mm^2^, the size of the matrix was set to 168 × 134, the number of the slice was set to 19, slice thickness was set to 5 mm, acquisition time was set to 23 s. For DSC-PWI (TR = 1,500 ms, TE = 30 ms, FOV = 22 cm, matrix = 128 × 128, 19 × 5 mm slices, total scan time = 1 min 38 s), gradient-echo planar imaging was performed during the passage of 0.1 mmol/kg of gadolinium-based contrast agent (Magnevist; Schering, Berlin, Germany) administered at a rate of 3 ml/sec. For each MRI image section, 50 temporal measurements were acquired for DCS-PWI analysis.

Valid MRI scanning images from 104 patients with carotid stenosis were included in this paper.

### 2.4. Radiomic-based ML as assessment model

#### 2.4.1. Plaque segmentation, data processing and feature extraction

Two board-certified radiologists were invited to analyze all images, with eight and five years of imaging experience in clinical practice. ROIs were obtained by manually segmenting SPACE images using 3D-Slicer (version 5.0.3). The segmentation label of each image is fulfilled by one radiologist and checked by the other.

Due to the limited amount of training data in our dataset, volumentations (19) techniques including “Random Rotation”, “Random Flip”, “Gaussian Blur”, “Gaussian Noise” and four combinations of static data augumentations were used to expand the dataset to sixty times the original dataset, which could also help the model to focus on task-related features (20). The augmented data were used as train data only and were not used in the model testing. Radiomics features were extracted using the pyradiomics (21) feature package based on Anaconda Prompt (version 4.2.0), according to the Image Biomarker Standardization Initiative (IBSI) feature guidelines. All images were co-registered, normalized, interpolated, and resampled to 1 × 1 × 1 mm^3^ resolution prior to radiomics extraction. First-order features (e.g., energy, entropy and mean), shape features (e.g., sphericity, surface area, voxel volume, etc.), gray level cooccurrence matrix (GLCM), gray level run length matrix (GLRLM), gray level size zone matrix (GLSZM) and gray level dependence matrix (GLDM) of original images and filtered images were extracted by pyradiomics.

#### 2.4.2. Radiomic feature selection

We chose four methods to select radiomics features. One method did not involve feature selection, while the other three methods used feature selection. The three methods were LASSO, ANOVA_LASSO, and ANOVA_spearman_LASSO. LASSO employed only the LASSO feature screening method, while ANOVA_LASSO used the ANOVA feature selection method first, followed by the LASSO feature screening method. ANOVA_spearman_LASSO utilized the ANOVA feature screening method first, followed by the spearman correlation coefficient screening, and finally, LASSO for feature selection.

In this paper, we present ANOVA_spearman_LASSO as an example and provide technical details for its three steps. Other feature selection methods follow the same pattern. Firstly, we calculated the ANOVA (One Way Analysis of Variance) *p*-values between labels and features in the classification task and removed features with *p* > 0.05. In this step, we followed the first step of radiomics features selection in Yang et al.’s paper (22). Secondly, to construct radiomics features, similar features with high correlation were rejected using Spearman's correlation analysis. Feature pairs with a Spearman's correlation coefficient greater than 0.9 were considered highly correlated features, and only one type of feature was used in the feature engineering. Finally, the Least Absolute Shrinkage and Selection Operator (LASSO) method (23) was used to select the features with non-zero coefficients from the primary dataset. The features selected by LASSO were normalized using the Z scores in the training and validatation datasets.

#### 2.4.3. Radiomics-based ML approach as assessment model

The radiomics-based ML approach used nine classic ML models: K-Nearest Neighbor (KNN), Logistic Regression (LR), Support Vector Machines (SVM), Decision Tree (DT), Random Forest (RF), XGBoost, AdaBoost, LightGBM, CatBoost, Multilayer Perceptron (MLP). These algorithms were implemented using Scikit-learn, an open-source Python ML library (24). In order to obtain optimal hyperparameters, grid search optimization with repeated stratified five-fold cross-validation was used to fine-tune the models to reduce bias due to model overfitting. The hyperparameters with the highest average AUC score during the five-fold cross-validation were considered the best model for this particular round of five-fold cross-validation analysis. The five-fold cross-validation returns with only one set of optimal hyperparameters. Five repetitions of five-fold cross-validation result with five different configurations of data segmentation. Therefore, there are five different sets of hyperparameters for the repeated five-fold cross-validation. The range of hyperparameters to find using the grid search method and its description can be found in the Supplementary S2. Similar technologies were also used in the following analysis to evaluate the models generated via the Deep Learning approach.

### 2.5. Deep learning approach as assessment model

DL techniques, especially convolution neural networks, have demonstrated outstanding performance in diagnostic and image analysis tasks (25). In contrast to traditional ML methods, they do not require quantification and selection of radiological features, as they are trained directly on the image in an end-to-end paradigm. In this study, two different network architectures were trained and evaluated, including 3D-DenseNet (26) and 3D-SE-DenseNet (13).

The three-dimensional rectangles are padded from the ROI annotation. After assessing the general size range of carotid arteries, we determined a 3D rectangular cube with a size of 30 × 30 × 60 pixels.

The multilayer perceptron, 3D-DenseNet and 3D-SE-DenseNet choose the same optimiser and share the same set of hyperparameters. A stochastic gradient descent (SGD) optimizer was used to minimize the cross-entropy loss between the model output and the target classification labels. A weighted random sampler was used to overcome the sample imbalance problem in this study. We utilized the same static data augmentation method as the previously mentioned radiomics-based machine learning method. In deep learning, a grid search method is used to find the best learning rate and batch size parameters for optimal performance. The range of hyperparameters in deep learning methods can be found in the Supplementary S2. We stopped training after 500 epochs. A repeated stratified five-fold cross-validation technique was used and the model with the optimal stratified cross-validation evaluation metric was obtained using the same hyperparameters. The classification performance of all two network architectures was tested in the same way. We evaluated the DL models by accuracy, precision, recall, F1 score, and area under the curve (AUC). A decision curve analysis was conducted to further assess the classification models.

#### 2.5.1. DenseNet

**Figure 2:**
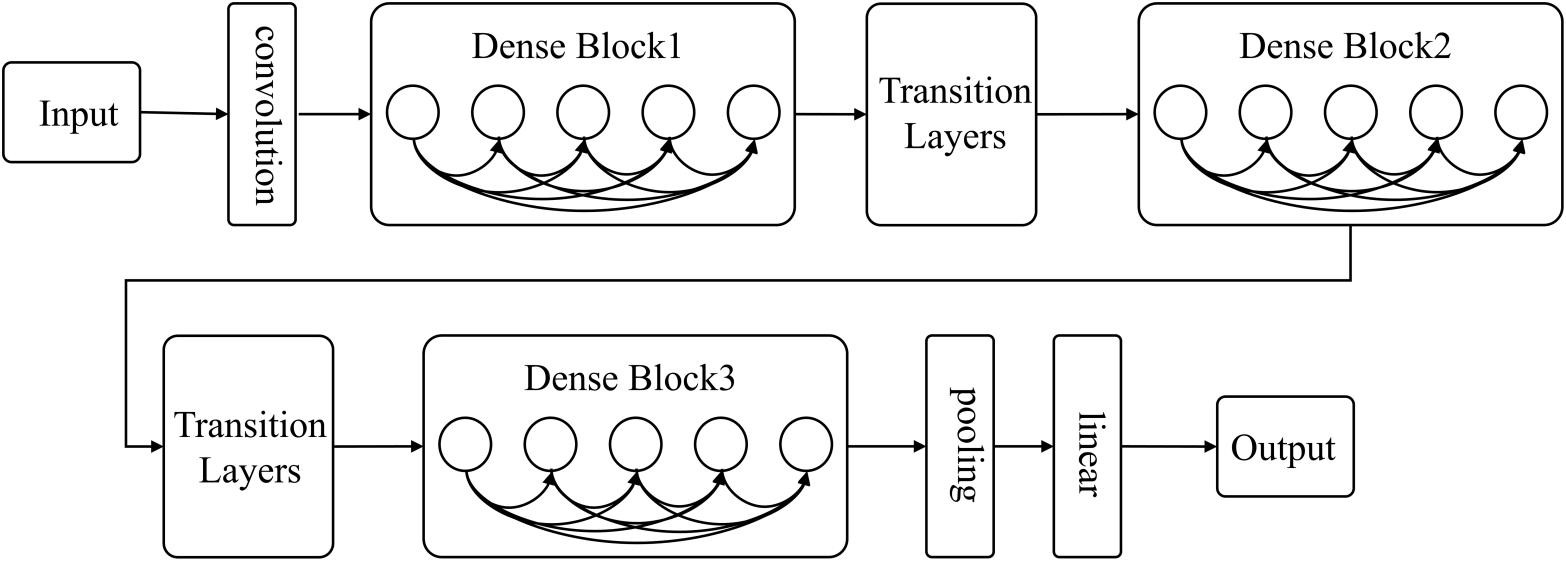
DenseNet network structure diagram.

As deep learning networks get deeper, the issue of gradient disappearance becomes increasingly apparent. This is where DenseNet comes in, improving on other networks by reducing the number of parameters and addressing gradient disappearance. The network links all layers to the feature map directly to ensure maximum information transmission. In contrast to traditional convolutional neural networks, where L connections exist in the L layer, DenseNet boasts L(L + 1)/2 connections. Alternating between Dense Block and Transition layers (As shown in Figure 2), the Dense Block is crucial to the structure, connecting every layer in the network and promoting information transfer while reducing gradient disappearance. This reduces the number of parameters and makes the network simpler to train. Transition layers sit between Dense Blocks and contain a batch normalization layer (BN), convolution layer (Conv), and an average pooling layer to further lessen dimensions.

#### 2.5.2. SENet

SENet is a sub-network structure that enhances network performance at the feature channel level. By automatically determining the importance of each feature channel through learning, SENet improves useful features and suppresses those that are not as useful for the task at hand. The network comprises Squeeze, Excitation, and Reweight blocks.

**Figure 3:**
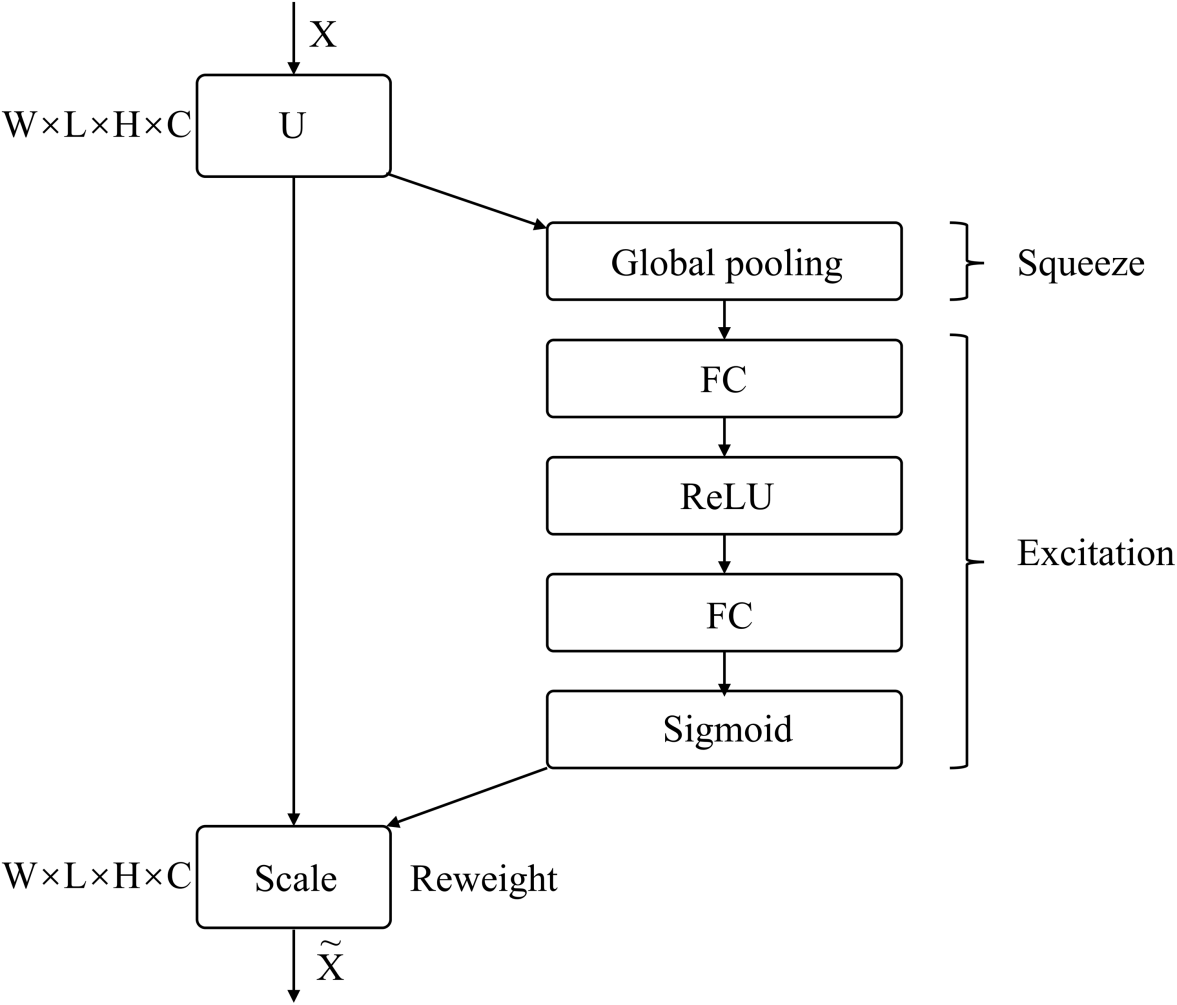
SE block structure.

Figure 3 depicts the SE Block, where X represents the input image with c1 feature channels. A series of convolution operations on X yields U with c2 feature channels. Through the Squeeze operation, the entire network acquires a global receptive field, while the Excitation generates weights for each feature channel. Finally, the Reweight re-calibrates original features in the channel dimension, to create an operation from U to X.

#### 2.5.3. SE-DenseNet

**Figure 4:**
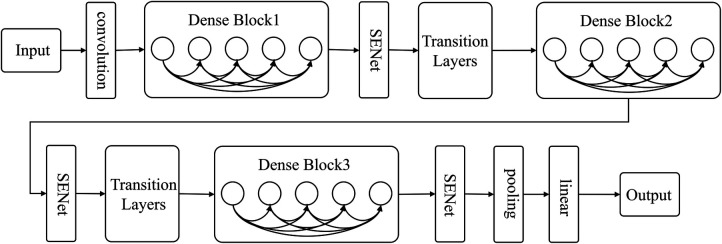
SE-DenseNet structure diagram.

The present research utilizes DenseNet's dense connection and SENet's feature recalibration feature to classify carotid plaque. A sub-structure network, SENet, is incorporated into DenseNet to create SE-DenseNet, as illustrated in Figure 4. By placing SENet before and after each Dense Block in the network, SE-DenseNet can effectively obtain and enhance beneficial features while suppressing features that are not relevant to the current task.

### 2.6. Evaluation metrics

To evaluate our models with an unbalanced sample size, we used the AUC value as the primary indicator. We represented the relationship between the recall rate and false positive rate at different decision thresholds through the generation of an ROC curve. The AUC, a dependable metric for evaluating model classification performance, was derived from the ROC curve. A score of 1.00 denotes perfect separation, while a score of 0.50 corresponds to random classification.

In addition to AUC, we utilized other measures to assess the models’ performance. Accuracy represents the model's robustness and is defined by the percentage of correctly identified labels out of the entire population. Precision, or the positive predictive value, is the probability that a predicted true label is indeed true. Sensitivity, referred to as the true positive rate (TPR) or recall, is the percentage of correctly identified true class labels. Lastly, the F1-score, which is the harmonic mean of sensitivity and precision, was used as another measure.In the result section, the results are shown as the mean of repeated five-fold cross-validation.

### 2.7. Statistical analysis

The Means ± SDs were calculated for continuous variables and percentages for categorical variables. For analysis of variance, we included variables that showed statistical significance in the one-way ANOVA test and variables with Spearman's correlation coefficient less than 0.9. All statistical analysis were performed using SPSS 24.0. Two-sided *p*-value of <0.05 was considered statistically significant.

## 3. Results

We provide a detailed evaluation and variance of radiomics-based ML and DL models using repeated stratified five-fold cross-validation approach in the Supplementary S1.

### 3.1. Demographics

**Figure 5:**
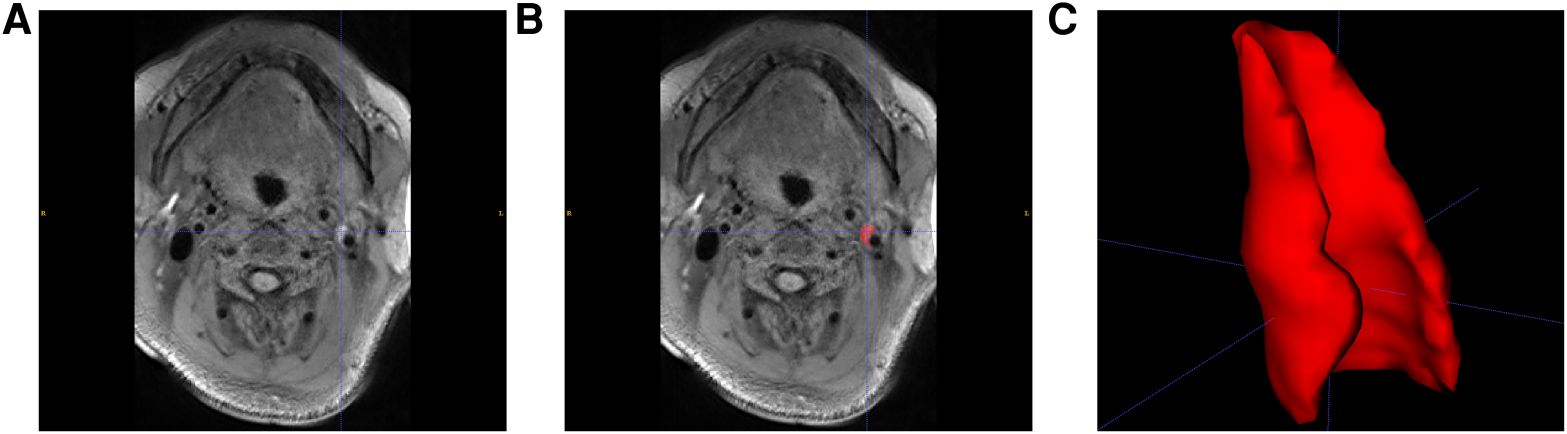
Carotid plaque segmentation. (**A**) SPACE sequence of MRI images with a carotid plaque in a patient. (**B**) Manually selected plaque segmentation. (**C**) 3D reconstruction of carotid plaque.

MRI imaging data from 104 patients (86 males and 18 females) with carotid stenosis were included in the analysis. 22 patients' imaging data were acquired from the Tianjin First Central Hospital and 82 patients' imaging data were acquired from the Tianjin Huanhu Hospital, with a median age of 64 years (range: 41–82). The demographics were shown in Table 1. 74 of the 104 patients were diagnosed with ischemic stroke. Figure 5 showed images of carotid plaque in a representative case of carotid stenosis.

**Table 1:** Demographic and imaging marks of patient populations.

Characteristic	Normal (*n* = 74)	Stroke (*n* = 30)
Age (mean ± SD[years])	64.61 ± 7.46	64.90 ± 7.31
Male	59	27
Female	15	3
ADC <620 × 10^−6^ mm^2^/s (mean ± SD[ml])	0 ± 0	0.86 ± 2.54
Tmax >4 s (mean ± SD[ml])	28.34 ± 81.88	74.54 ± 115.41
Tmax >6 s (mean ± SD[ml])	1.02 ± 3.24	53.48 ± 38.26
Tmax >8 s (mean ± SD[ml])	0.63 ± 3.49	2.54 ± 6.61
Tmax >10 s (mean ± SD[ml])	0.14 ± 1.20	0.31 ± 1.71

### 3.2. Radiomics features for the carotid plaques

**Figure 6:**
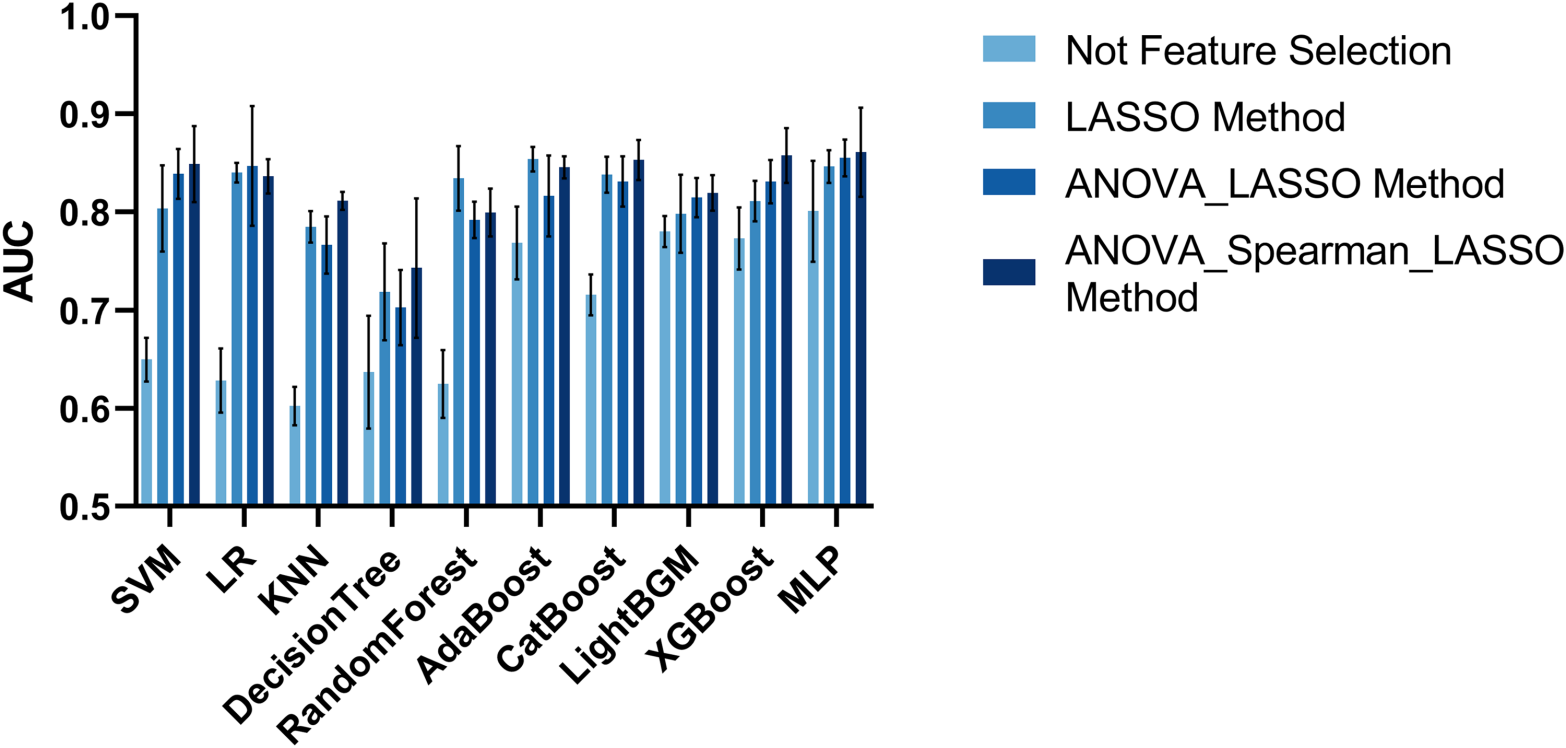
The AUC of the radiomics-based ML model with four feature selection methods.

In total, 5,174 features were initially extracted from the SPACE of MRI. We utilized four methods to select radiomics features. One approach for constructing a classification model in radiomics does not require feature selection as it utilizes the complete set of radiomics features available. The AUC metrics of the classification models are shown in Figure 6. The model with the best classification performance is the Multilayer Perceptron model. The AUC, accuracy, precision, sensitivity, and F1 score of Multilayer Perceptron are 0.8009, 0.7824, 0.5994, 0.5568, and 0.5892 respectively.

The AUC evaluation metrics of the classification model using the LASSO feature selection method are shown in Figure 6. The model with the best classification performance is the Multilayer Perceptron model. The AUC, accuracy, precision, sensitivity, and F1 score of Multilayer Perceptron are 0.8465, 0.8326, 0.7849, 0.6462, and 0.6735 respectively.

The AUC evaluation metrics of the classification model using the ANOVA_LASSO feature selection method is shown in Figure 6. The model with the best classification performance is the Multilayer Perceptron model. The AUC, accuracy, precision, sensitivity, and F1 score of Multilayer Perceptron are 0.8552, 0.8130, 0.6686, 0.6859, and 0.6699 respectively.

The AUC evaluation results of the classification model using the ANOVA_spearman_LASSO feature selection method are shown in Figure 6. The model with the best classification performance is the Multilayer Perceptron model. The AUC, accuracy, precision, sensitivity, and F1 score of Multilayer Perceptron are 0.8611, 0.8189, 0.7965, 0.7783, and 0.7525 respectively.

The classification performance of radiomics-based ML approaches in discriminating SPs and ASPs based on the best feature selection approach, i.e., ANOVA_spearman_LASSO, was summarized in Table 2.

**Table 2:** The outcome of the radiomics-based ML models with ANOVA_spearman_LASSO method. Models with the highest performance are highlighted in bold.

Models	Accuracy (%)	Precision (%)	Sensitivity (%)	F1-score (%)	AUC (%)
SVM	80.36 ± 5.45	74.63 ± 3.58	71.50 ± 1.74	66.37 ± 3.69	84.89 ± 3.89
LR	80.77 ± 6.67	69.85 ± 2.43	64.48 ± 4.79	66.11 ± 6.26	83.63 ± 1.75
KNN	78.28 ± 8.05	66.45 ± 10.02	67.37 ± 2.85	70.06 ± 2.15	81.16 ± 0.92
Decision Tree	78.86 ± 8.00	72.66 ± 4.95	60.49 ± 8.89	72.57 ± 3.79	74.29 ± 7.10
Random Forest	79.04 ± 6.31	71.31 ± 2.01	71.31 ± 2.01	71.28 ± 3.11	79.95 ± 2.43
AdaBoost	81.73 ± 7.16	76.47 ± 5.30	68.18 ± 5.33	74.08 ± 4.59	84.57 ± 1.12
CatBoost	81.15 ± 6.63	79.73 ± 8.30	74.52 ± 2.70	73.72 ± 2.54	85.32 ± 2.03
LightGBM	80.58 ± 8.56	76.67 ± 5.44	74.55 ± 2.91	74.74 ± 3.70	81.95 ± 1.81
XGBoost	80.55 ± 6.20	71.89 ± 3.87	68.18 ± 5.60	73.99 ± 2.26	85.78 ± 2.79
MLP	**81.89 ** **± ** **7.03**	**79.65 ** **± ** **3.32**	**77.83 ** **± ** **4.94**	**75.25 ** **± ** **3.38**	**86.11 ** **± ** **4.54**

**Figure 7:**
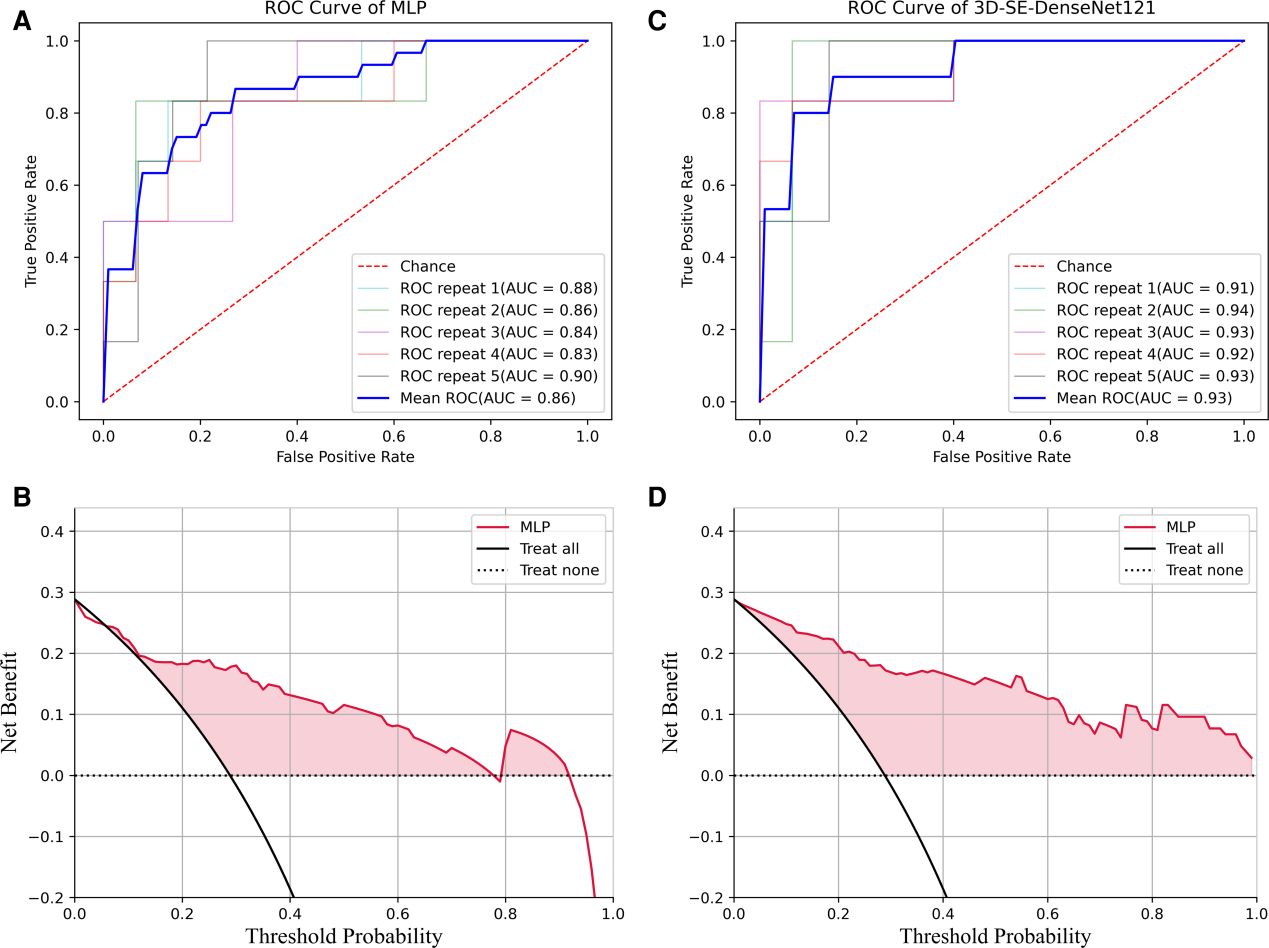
ROC and DCA results of the MLP and 3D-Densenet121. (**A**) ROC for MLP method; (**B**) DCA curve analysis for the best run of MLP method; (**C**) ROC for 3D-SE-Densenet121method; (**D**) DCA for the best run of 3D-SE-Densenet121 method.

Our results demonstrated that the radiomics-based ML model employing ANOVA_spearman_LASSO feature selection and MLP classification displayed the highest AUC value. The ROC curve for this model, as measured by repeated stratified five-fold cross-validation, is shown in Figure 7A.

### 3.3. The DL approach as assessment model

Two DL frameworks were used in this section, 3D-DenseNet, and 3D-SE-DenseNet, and several models were derived from the original framework and evaluated in this paper. The 3D-DenseNet model series were generated and assessed in the image dataset, including 3D-DenseNet121, 3D-DenseNet169, 3D-DenseNet201, 3D-DenseNet264. The 3D-DenseNet demonstrated the best performance. The 3D-SE-Densenet model series included 3D-SE-Densenet121, 3D-SE-Densenet169, 3D-SE-Densenet201 and 3D-SE-Densenet264. The 3D-SE-Densenet121 showed the best performance.

The performance of the CNN models in differentiating SPs and ASPs was shown in Table 3. The AUC, accuracy, precision, sensitivity, and F1 score of 3D-Densenet121 algorithm were 0.8968, 0.9094, 0.8795, 0.8035, and 0.8556, respectively. The AUC, accuracy, precision, sensitivity, and F1 score of 3D-SE-DenseNet121 algorithm were 0.9300, 0.9308, 0.9008, 0.8588, and 0.8614, respectively. Based on these results, 3D-SE-DenseNet121 outperformed the 3D-DenseNet. The AUC, accuracy, precision, sensitivity and F1 score of the 3D-SE-DenseNet121 algorithm were 0.0332, 0.0214, 0.0213, 0.0553, and 0.0058 higher than those of 3D-DenseNet121, respectively.

**Table 3:** The outcome of the deep learning models. Models with the highest performance are highlighted in bold.

Models	Accuracy (%)	Precision (%)	Sensitivity (%)	F1-score (%)	AUC (%)
3D-DenseNet121	90.94 ± 3.81	87.95 ± 5.02	80.35 ± 5.05	85.56 ± 5.85	89.68 ± 2.60
3D-DenseNet169	88.47 ± 4.43	82.18 ± 4.63	81.15 ± 6.11	80.20 ± 3.61	88.55 ± 4.59
3D-DenseNet201	87.88 ± 4.64	83.85 ± 5.03	80.04 ± 4.05	83.62 ± 3.96	85.77 ± 1.92
3D-DenseNet264	85.98 ± 4.56	80.43 ± 3.17	78.90 ± 1.95	79.27 ± 3.41	84.94 ± 3.49
3D-SE-DenseNet121	**93.08 ** **± ** **3.67**	**90.08 ** **± ** **3.38**	**85.88 ** **± ** **2.26**	**86.14 ** **± ** **4.28**	**93.00 ** **± ** **1.96**
3D-SE-DenseNet169	92.10 ± 3.13	87.77 ± 2.11	80.51 ± 8.41	82.80 ± 2.70	91.27 ± 1.58
3D-SE-DenseNet201	88.85 ± 3.32	82.45 ± 4.26	82.41 ± 2.47	79.63 ± 3.78	87.76 ± 2.11
3D-SE-DenseNet264	85.61 ± 5.95	81.76 ± 1.32	80.96 ± 3.25	84.21 ± 3.03	86.97 ± 2.07

The ROC curve for 3D-SE-DenseNet121, as measured by repeated stratified five-fold cross-validation, is shown in Figure 7C.

### 3.4. Decision curve analysis

We performed a decision curve analysis (DCA) to assess the classification models in terms of net benefit against threshold probability, which could be crucial in clinical applications. A decision curve analysis graph with threshold probability on the x-axis and net benefit on the y-axis illustrated the trade-offs between true positives and false positives as the threshold probability varies, respectively.

Figure 7B showed the DCA results for the best run of the best-performing ML model using MLP. The decision curve analysis for the best run of MLP showed that within a threshold probability from 6% to 77% or 80% to 91%, checking patients based on the classification model leads to a higher net benefit than assigning all patients as SPs or ASPs.

To optimize the 3D-SE-Densenet121 model, DCA analysis (Figure 7D) suggests that utilizing threshold probabilities between 1% to 99% would yield the most significant advantages for the classification model.

Comparing the DCA results of 3D-SE-Densenet121 and MLP, the 3D-SE-Densenet121 demonstrated more robust performance and brought more net benefit than MLP with a wider range of threshold probability.

## 4. Discussion

In this study, we recruited 104 patients with carotid stenosis. HRMRI was conducted to acquire imaging data. The radiomics-based ML approach and DL approaches were proposed and investigated in this study to differentiate SPs and ASPs.

The ANOVA_spearman_LASSO and MLP model combination has emerged as the most effective radiomics-based ML model, as shown by our research. Utilizing feature selection, the best radiomics-based ML models demonstrated superior performance, with higher AUC, accuracy, precision, sensitivity, and F1 scores than models without feature selection, with differences of 0.0602, 0.0365, 0.1971, 0.2215, and 0.1633, respectively. These findings underscore the significance of feature selection in accurately distinguishing between ASPs and SPs. By implementing feature selection in our study, we have gained numerous benefits. Firstly, it has enhanced the accuracy of our model and mitigated the danger of overfitting. Moreover, feature screening has deepened our comprehension of the model's workings and has also reduced computational costs.

By utilizing ANOVA_spearman_LASSO, the top radiomics-based ML models achieved superior performance as compared to models utilizing ANOVA_LASSO. The former displayed significantly higher AUC, accuracy, precision, sensitivity, and F1 scores, with differences of 0.0059, 0.0059, 0.1279, 0.0924 and 0.0826, respectively. These results underscore the significance of utilizing the spearman method in accurately distinguishing between ASPs and SPs. The MRI image data is amplified by sixty-fold using static data enhancement techniques. Radiomics technique yielded 5,174 extracted image histology features. However, some of these features, such as the first-order features in the “random rotate” approach to data enhancement, were redundant. To address this issue, we employed the Spearman method, a nonparametric, ranking-based method that is better equipped to handle nonlinear data relationships, eliminate unimportant and irrelevant features, and prevent overfitting and underfitting of the model. As a result, the filtered features were more representative, better explained the prediction results.

The raw MRI image data is amplified by a factor of sixty through static data enhancement techniques. With the radiomics technique, we extracted a total of 5,174 image histology features. Although we extracted a large number of image histology features, we included a large number of redundant features, such as first order features in the “random rotate” approach to data enhancement. The Spearman method is a nonparametric, ranking-based method that can better handle the nonlinear relationships of the data, eliminate unimportant and useless features, and avoid overfitting and underfitting of the model. The filtered features are more representative, can better explain the prediction results of the model, and are more easily understood by people.

The 3D-SE-Densenet121 showed the best performance among all models. The best performance method for radiomics-based ML approach was the combination of ANOVA_spearman_LASSO and MLP. The best performance method for the DL approach was 3D-SE-Densenet121 model. The AUC, accuracy, precision, sensitivity, and F1 scores of the best DL method (3D-SE-Densenet121) were 0.0689, 0.1119, 0.1043, 0.0805, and 0.1089 higher than those of the best radiomics-based ML models (MLP), respectively.

It was clear that the DL models had better performance than the radiomics-based ML model in differentiating ASPs from SPs (AUC = 0.9294 vs. AUC = 0.8853). These results were consistent with the findings for Mantle Cell Lymphoma (27) and Deep Vein Thrombosis (28) that DL models had better diagnostic performance than radiomics-based ML models. This was due to the fact that DL extracts more representative high-level abstract features from the raw data, while machine learning requires manual feature selection and design. In addition, it was clear that the model performance was directly reflected in the DCA results. The 3D-SE-Densenet-121 model demonstrated the highest performance in all the model evaluation metrics. Unlike the MLP model based on radiomics features, the 3D-SE-Densenet-121 model demonstrated stable and robust net benefit in DCA. Furthermore, the LightGBM, XGBoost, Multilayer Perceptron models and the CNN-based 3D-SE-Densenet model (i.e., nonlinear classifiers with high complexity) showed higher performance compared to other models, which suggested that models with higher nonlinear complexity were favored for HRMRI data.

Additionally, compared with the AUC results of other papers, our classification models for SPs and ASPs have achieved better performance. Li et al. (10) constructed a 3D HRMRI-based radiomics model to identify symptomatic plaques with an AUC of 0.906. Compared to our study, Li et al.'s study had a lower AUC than our best model's 0.9300, and he used a single-center dataset while we acquired data from two centers. Huang et al. (29) used radiomics ultrasonography to non-invasively predict SPs and ASPs with a training set of 0.930 and a test set of 0.922, which is also lower than the AUC of our highest model. The two-dimensional radiomics model using maximum plaque area slices to classify carotid plaques in the study by Zhang et al. (9) showed better performance than the conventional methods (AUC = 0.984 vs. AUC = 0.804), but the size of the dataset and the absence of repeated stratified cross-validation might lower the reproducibility of this paper.

Although, this paper was not intended to focus on model complexity, the analysis of the relationship between the number of learning parameters and model performance was not included in this paper, we still observed some preliminary performance discrepancies with different number of learning parameters and different network architectures. Among all the models tested in this paper, our experiments showed that 3D-DenseNet121, which has the fewest parameters among the four 3D-DenseNet models, demonstrated the best performance. Similarly, 3D-SE-DenseNet121, which was the 3D-SE-DenseNet model with the fewest parameters among the four 3D-SE-DenseNet models, yielded the best results in our experiments. Furthermore, 3D-SE-DenseNet121 model outperformed 3D-DenseNet despite that 3D-SE-DenseNet121 had more parameters. These results suggests that the SE Block has a positive impact on the model's performance. Therefore, when working with small spatial sample sizes, choosing the right number of parameters and architecture was crucial.

To our knowledge, this paper represents the first study to utilize 3D HRMRI-based radiomics-based ML and DL models for evaluating carotid plaque properties. Through comparing and systematically examining these two approaches with the same dataset, our proposed methods have demonstrated robustness and high-performance, particularly in the case of the DL approach.

However, our study has several limitations. First of all, the limitation in the size of the MRI image data set severely limits the robustness and generalizability of the findings in this paper, future studies should be conducted with larger cohorts to further validated the methods proposed in this paper. Limited by the scale of our research and restricted availability of clinical data, it is clear that the models' performance in this paper are still far from practical applications in clinical scenarios. Larger datasets are thus required for further investigation of the models from diverse perspectives to investigate their clinical efficacy. Secondly, future studies with fully automated segmentation and classification methods should be investigated to further streamline the analysis and minimize intervention from the healthcare professionals. In the end, although radiomics methods seem to be easier to interpret in terms of the significance of features, there are several methods to explain DL models. For example, local saliency analysis, class activation mapping and other methods can be used to investigate the features learned by the DL models which may further broaden our understanding in the imaging characteristics of SP plaques.

## 5. Conclusion

In this paper, a multi-center high-resolution carotid MRI dataset was constructed, and radiomics-based ML and DL approaches were evaluated for the classification of carotid plaques. Compared with radiomics-based ML approaches, DL approaches demonstrated superior performance in the classification of carotid plaques on sequential HRMRI, especially the 3D-SE-Densenet models, in terms of accuracy and AUC. The 3D-SE-Densenet-121 model showed the best performance among all models.

## Data Availability

The raw data of this paper were patient imaging data and our ethics restrict publishing/sharing raw data to any other institutions. However, the scripts and codes can be available upon requests.
