# Supplementary material for: Radiomics and artificial neural networks modelling for identification of high-risk carotid plaques
Source: Front Cardiovasc Med. 2023 Jul 6;10:1173769. doi: 10.3389/fcvm.2023.1173769 (PMC10358979; doi:10.3389/fcvm.2023.1173769)
Supplement: Supplementary file 2 [file Datasheet2.docx]

Supplementary Material

Radiomics and Artificial Neural Networks Modelling for Identification of High-risk Carotid Plaques

**Chengzhi Gui^1,2#^, Chen Cao^3#^, Jiaxin Zhang^4^，Xin Zhang^1,2*^, Guangjian Ni^1^, Dong Ming^1^**

^1^Academy of Medical Engineering and Translational Medicine, Tianjin University, Tianjin, China

^2^Institute of Biomedical Engineering, Chinese Academy of Medical Sciences and Peking Union Medical College, Tianjin, China^3^Department of Radiology, Huanhu Hospital, Tianjin University

^4^School of Medical Science and Engineering, Tianjin University, Tianjin, China

Institution^1^ and Institution^2^ contributed equally to this work.

^#^These authors contributed equally to this work

*Corresponding Author

# Supplementary Data

The ranges of the grid search is as follows.

SVM

'C': [1e-3, 1e-2, 1e-1, 1, 2, 5, 8],

'gamma': [0.001, 0.0001]

C: Penalty factor. C controls the degree of penalty for misclassified samples, and it is a regularization parameter.

gamma: kernel function coefficients. gamma controls the shape of the kernel function.

LR

"penalty": ['l1', 'l2']

'C': [i for i in np.linspace(0.05, 1, 19)]}

penalty: regularization method.penalty controls the intensity and manner of regularization during the training process.

C: Penalty coefficient.C has the same meaning as the penalty factor C in SVM, and is a penalty measure for the model in case of errors in the actual sample. A smaller C allows the model to generalize more, and a larger C allows the model to perform better on the training set.

KNN

"n_neighbors": [i for i in range(1, 11)]

n_neighbors: K-value. n_neighbors refers to the K-value in the KNN algorithm, which indicates the number of neighbors used for decision making.

DecisionTree

'criterion': ['gini',’entropy’]

'max_depth': [1, 2, 3, 5, 10, 20, 30, 50, 60, 100]

'min_samples_leaf': [1, 2, 3, 5, 10, 20],

'min_impurity_decrease': [0.001, 0.01, 0.1, 0.2, 0.5]}

criterion: splitting strategy.criterion specifies the function used to measure the quality of splitting. The main difference between decision trees is the difference in splitting strategies.

max_depth: the maximum depth of the tree.The max_depth parameter is used to control the maximum depth of the tree in order to prevent overfitting. If max_depth is set too large, the decision tree will be too complex and likely to overfit; if max_depth is set too low, it may not capture the complex structure of the data.

min_samples_leaf: leaf node sample count threshold.The min_samples_leaf parameter specifies the minimum number of samples that must be contained on a leaf node. If the number of samples on a leaf node is less than min_samples_leaf, the split will not be performed, which also prevents the tree from being split too thinly.

min_impurity_decrease controls the degree of node splitting. It indicates that the splitting stops when the overall impurity reduction from the splitting of a node is less than the value of this parameter.

RandomForest

'min_samples_leaf': range(1, 10, 3)

'min_samples_split': range(2, 11, 3)

'max_leaf_nodes': [None] + list(range(20, 70, 20))

'max_depth': range(5, 16, 5)

'max_samples': [None, 0.6, 0.5, 0.4]

'n_estimators': range(10, 160, 70)

'max_features': ['sqrt', 'log2'] + list(range(1, 6, 1))

min_samples_leaf: The minimum number of samples required for the leaf nodes of the decision tree. This parameter controls the number of leaf nodes.

min_samples_split: the minimum number of samples to split per decision tree node. Set this parameter to control the number of decision tree nodes.

max_depth: the maximum depth of the decision tree. Too much depth can easily lead to overfitting the model, while not enough depth can lead to underfitting the model.

max_samples is the number of samples used to construct each decision tree, which can be used to adjust the variance and bias of the decision tree.

n_estimators: the number of trees. It indicates the number of decision trees used in the construction of the random forest.

max_features: the number of features to be considered for each decision tree when splitting nodes. It can effectively reduce the risk of overfitting, but will reduce the prediction accuracy of the model.

AdaBoost:

'n_estimators': list(range(2, 102, 2))

'learning_rate': [0.1, 0.2, 0.3, 0.4, 0.5, 0.6, 0.7, 0.8, 0.9, 1]

n_estimators: the number of trees. It indicates the number of iterations, i.e. the number of weak classifiers.

learning_rate: The learning rate. It indicates how much each weak classifier contributes to the final classifier. When the learning rate is small, the contribution of each classifier to the final classifier will decrease; when the learning rate is large, the contribution of each classifier to the final classifier will increase.

CatBoost:

'depth': [4, 6, 10]

'learning_rate': [0.05, 0.1, 0.15]

'l2_leaf_reg': [1, 4, 9]

Depth: the maximum depth of the tree. The depth of the tree is the number of layers from the root node to the bottommost leaf node. Increasing the depth value increases the fitting ability of the model, which means it increases the complexity of the model and is able to learn the training data better.

learning_rate: The learning rate. It indicates how much each weak learner contributes to the final model. A smaller learning rate will make the model more robust, but may increase the time it takes for the model to converge.

l2_leaf_reg: L2 regularization parameter. CatBoost uses L1 and L2 regularization to reduce overfitting, where L2 regularization is the addition of a weight squared term to the loss function to reduce some of the weight parameters and thus suppress overfitting.

LightBGM

'learning_rate': [0.01, 0.1, 1]

'n_estimators': [10, 20, 40, 50, 100]

learning_rate: The learning rate. It represents the extent to which each weak learner contributes to the final model.

n_estimators: the number of weak learners. Each time a weak learner is added, the model is optimized once, making the model's fitting ability gradually improve.

XGBoost

'max_depth': range(2, 7)

'n_estimators': range(100, 1100, 200)

'learning_rate': [0.05, 0.1, 0.25, 0.5, 1.0]

max_depth: the maximum depth of the tree. It helps us to control the complexity and generalization ability of the model. A smaller max_depth may lead to overfitting and a larger max_depth may lead to too high model complexity.

n_estimators: the number of weak learners. Each time a weak learner is added, the model is optimized once, making the model's fitting ability gradually improve.

learning_rate: The learning rate, which controls how much the weights are reduced after each iteration of the update. When the learning rate is small, the algorithm requires more iterations to converge, but the results are usually more accurate.

3D-DenseNet121, 3D-DenseNet169, 3D-DenseNet201, 3D-DenseNet264, 3D-SE-DenseNet121, 3D-SE-DenseNet169, 3D-SE-DenseNet201, 3D-SE-DenseNet264

learing_rate：[0.001, 0.0005, 0.0001, 0.00005]

batchsize: [2, 4, 8, 16]

learning rate: The learning rate is a hyperparameter in deep learning that controls the size of the update made to the model weights at each iteration of the optimization algorithm. It determines how quickly or slowly the model learns from the data.

batchsize: batchsize refers to the number of samples processed by the model in one training step.
